# Pre-Existing Allergic Inflammation Alters Both Innate and Adaptive Immune Responses in Mice Co-Infected with Influenza Virus

**DOI:** 10.3390/ijms26083483

**Published:** 2025-04-08

**Authors:** Dan Li, T. Anienke van der Veen, Linsey E. S. de Groot, Marina H. de Jager, Andy Lan, Hoeke A. Baarsma, René Lutter, Kees van der Graaf, Reinoud Gosens, Martina Schmidt, Barbro N. Melgert

**Affiliations:** 1 Groningen Research Institute of Pharmacy, Department of Molecular Pharmacology, University of Groningen, 9713 AV Groningen, The Netherlands; danli_emma@outlook.com (D.L.); avdveen4rug@gmail.com (T.A.v.d.V.); m.h.de.jager@rug.nl (M.H.d.J.); andy.lan@student.uts.edu.au (A.L.); h.a.baarsma@rug.nl (H.A.B.); r.gosens@rug.nl (R.G.); m.schmidt@rug.nl (M.S.); 2 Groningen Research Institute for Asthma and COPD (GRIAC), University Medical Center Groningen, University of Groningen, 9713 AV Groningen, The Netherlands; 3 Department of Respiratory Medicine, Amsterdam UMC, University of Amsterdam, 1105 AZ Amsterdam, The Netherlandsr.lutter@amsterdamumc.nl (R.L.); 4 Citeq Biologics BV, 9726 GN Groningen, The Netherlands; vandergraaf@citeq.com

**Keywords:** asthma, house dust mite, allergy, Th1 cells, Th2 cells, neutrophils

## Abstract

Asthma, a chronic airway disease, is marked by allergic inflammation, hyperresponsiveness, and tissue remodeling. Influenza infections in asthma patients can cause severe exacerbations, though the underlying mechanisms remain unclear. This study investigated how pre-existing allergic inflammation affects immune responses to influenza infection in mice exposed to house dust mite (HDM). Mice were repeatedly exposed to HDM, followed by infection with the influenza A virus, and were sacrificed three days post-infection. Plasma was analyzed for HDM-specific immunoglobulins, while lung tissue was used for immune cell flow cytometry and RNA sequencing analysis. HDM exposure induced allergic inflammation, evidenced by more HDM-specific IgE, IgG1, IgG2, eosinophils, neutrophils, Th1, and Th17 cells compared to controls. Upon influenza infection, the effects of HDM and influenza co-infection interacted, showing fewer Th1 cells and regulatory T cells and more Th2 cells compared to mice exposed to the influenza virus alone. Interestingly, RNA-seq analysis revealed less upregulation of Th1-related genes and antiviral pathways in co-exposed mice, suggesting impaired Th1 immunity and antiviral responses. Pre-existing allergic inflammation significantly altered immune responses in mice co-infected with influenza, revealing underdeveloped antiviral responses as early as three days post-infection. These findings may explain the increased susceptibility of patients with asthma to severe viral diseases.

## 1. Introduction

Allergic asthma, a globally prevalent non-communicable disease, impacts individuals of all ages. The disease presents with symptoms such as wheezing, coughing, chest tightness, and shortness of breath [1,2]. Patients often experience episodes of acute worsening of their asthma symptoms, known as exacerbations, frequently triggered by external factors, such as respiratory viral infections. In some cases, exacerbations can be limited and controlled with medication, but in others they lead to hospitalization and even death [3]. Among the causes of asthma exacerbations, the influenza virus is one of the most common, especially in adults [4,5].

**Figure 1:**
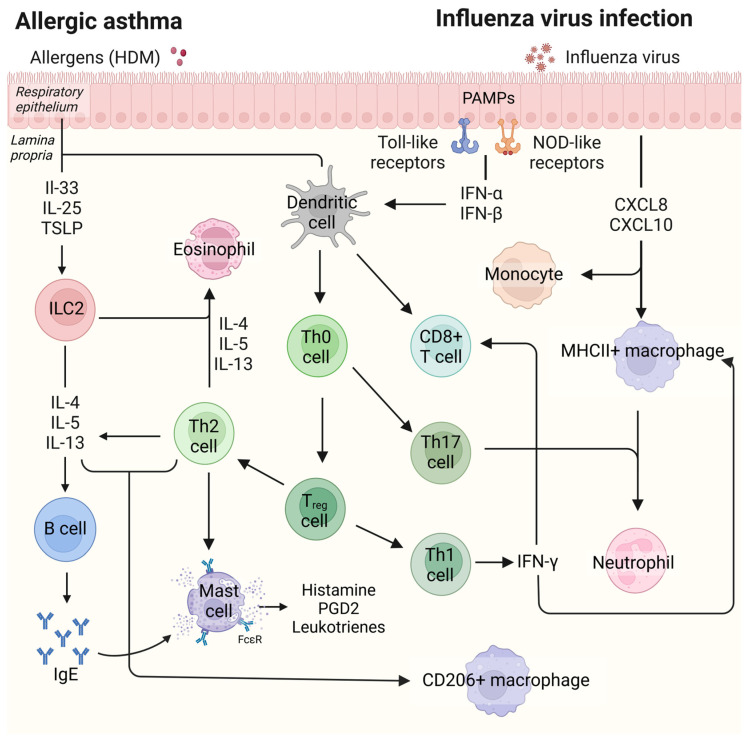
**Immune responses of allergic asthma and influenza virus infection.** Immune responses in allergic asthma involve Th2-driven mechanisms characterized by cytokines such as IL-4, IL-5, and IL-13, as well as higher serum IgE levels, eosinophil, and CD206+ macrophage counts in lung tissue; immune responses of influenza virus infection include Th1-mediated mechanisms characterized by increased levels of type I interferon and type III interferon, activation of neutrophils, MHCII+ macrophages, and CD8+ T cells. Abbreviations: CD, cluster of differentiation; CXCL8, C-X-C motif chemokine ligand 8; CXCL10, C-X-C motif chemokine ligand 10; HDM, house dust mite; IFN-α, interferon alpha; IFN-β, interferon beta; IFN-γ, interferon gamma; IgE, immunoglobulin E; ILC2, type 2 innate lymphoid cells; IL-4, interleukin-4; IL-5, interleukin-5; IL-13, interleukin-13; IL-25, interleukin-25; IL-33, interleukin-33; MHC II+, major histocompatibility complex type II; NOD-like receptors, Nucleotide-binding oligomerization domain-like receptors; PAMPs, pathogen-associated molecular patterns; PGD2, prostaglandin D2; Th0 cell, naive T cell; Th1 cell, type 1 T helper cell; Th2 cell, type 2 T helper cell; Th17 cell, type 17 helper cell; Treg cell, regulatory T cell; TSLP, thymic stromal lymphopoietin.

The underlying biology of allergic asthma involves a T helper 2 (Th2)-driven inflammation started by airway epithelial cells releasing alarmins (TSLP, IL-33, IL-25) in response to allergens (see Figure 1) [6]. This activates dendritic cells and innate lymphoid cells (ILCs) that drive adaptive immune responses [6,7]. ILCs produce type 2 cytokines (IL-4, IL-5, IL-13), promoting (Th2) cell responses [7]. Th2 cells secrete the same cytokines, leading to IgE production by B cells, activation of mast cells and eosinophils, alternative macrophage activation, increased mucus production, airway hyperresponsiveness, and airway remodeling [8,9,10]. In addition to Th2 cells, Th1 and Th17 cells may contribute to varying degrees, leading the recruitment of neutrophils [11]. Furthermore, dysregulated regulatory T cells (Tregs) have been shown to fail to suppress excessive inflammation, worsening asthma symptoms [12].

In contrast, immune responses to influenza are substantially different. In this case, airway epithelial cells detect viral antigens through pathogen-sensing receptors such as toll-like receptors [13] that activate transcription factors such as interferon regulatory factors and nuclear factor kappa-B to release type I interferons (IFN-α, IFN-β) and pro-inflammatory cytokines (e.g., TNF-α) to inhibit viral replication [14]. Epithelial cells also release chemokines (e.g., CXCL8, CXCL10) to recruit neutrophils and monocytes that help to limit the infection, while classically activated resident macrophages (major histocompatibility complex type II (MHC II+) macrophages) produce cytokines and reactive oxygen species to combat the virus [15]. During progression of these immune responses, a subset of macrophages transitions into an alternatively activated phenotype (CD206+ macrophages), releasing anti-inflammatory cytokines (e.g., IL-10, TGF-β) and growth factors and promoting tissue repair and the resolution of inflammation [16]. Lung-resident dendritic cells, activated by epithelial cell-derived chemokines (e.g., CCL2, type I/III interferons) [15], induce the development of influenza-specific CD4+ and CD8+ T cells. The latter kill infected cells while influenza-specific Th1 cells produce IFN-γ to enhance CD8+ T cell function [17]. Together, these innate and adaptive responses aim to clear the infection as efficiently as possible.

The interaction between an altered lung environment due to asthma and a subsequent influenza infection potentially alters infection outcomes, although the precise mechanisms remain to be fully elucidated. Previously, we have shown that changes in early innate immune responses may drive more severe viral disease in house dust mite (HDM)-exposed mice [18]. However, others have also shown opposite outcomes or few changes [19,20,21,22,23,24,25]. Most of these studies have demonstrated that mice with HDM-induced lung inflammation indeed experience more severe viral disease, characterized by more weight loss and higher viral titers, than mice without pre-existing allergic lung inflammation. However, it is difficult to establish from these studies exactly how the effects of HDM exposure and influenza infection interact during early infection. This is due to a combination of factors, including differences in exposure models, time points, and/or outcome parameters used and missing control groups to exactly establish interactions between HDM exposure and influenza infection. To address these gaps, we therefore designed a study specifically investigating early infection events in both mice with and without pre-existing HDM-induced inflammation. By combining whole-genome transcriptome analysis of lung tissue with a comprehensive flow cytometric analysis of both innate and adaptive immune responses, we aimed to characterize how both exposures interact in the early responses to influenza, particularly three days after infection. Based on the available data from previous studies, we expected most changes to occur in the presence of innate immune cells and viral response pathways and less so in HDM-induced adaptive immune responses. Surprisingly, however, our data clearly showed that adaptive responses, particularly Th1 infiltration, were also already altered three days after infection, possibly explaining why asthmatics are at risk for more severe viral disease when infected with influenza.

## 2. Results

### 2.1. Both Influenza Infection and HDM Exposure Result in Weight Loss, and Animals Exposed to Both Lose the Most Weight

**Figure 2:**
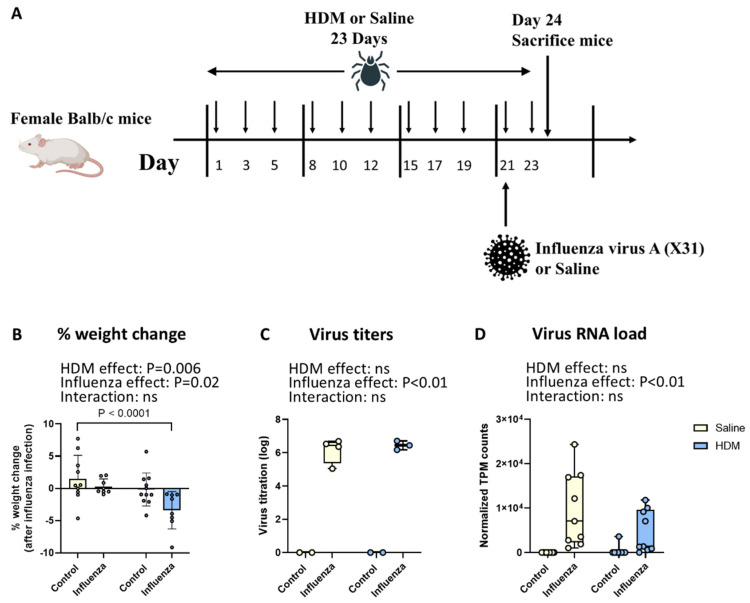
**Effect of HDM, influenza infection, and their combination on weight change, influenza titer, and the presence of influenza RNA in lung tissue.** Schematic of mice treated with house dust mite (HDM) and influenza virus A (X31) or saline (**A**). Percentage weight change after influenza infection for three days (**B**), data are presented as mean ± SD. Influenza virus titers (**C**) and normalized counts of virus RNA (**D**) in lung tissue, data are presented as medians, with range from minimum to maximum. Groups were compared using a two-way ANOVA; a one-way ANOVA was used for post hoc analyses in panel A; *p* < 0.05 was considered significant; ns, not significant.

Previous studies showed more severe disease in animals exposed to both HDM and influenza compared to those exposed only to influenza, resulting in more weight loss [19,21]. To investigate this and test our hypothesis, we exposed mice to saline or HDM three times per week for 24 days and a single, nasal administration of 20 units of 50% tissue culture infectious dose of the H3N2 influenza A virus HK X31 or saline on day 21, as described in Figure 2 (Figure 2A). The mice in our model lost a little weight when exposed to either HDM or influenza (*p* = 0.006 for HDM and *p* = 0.02 for influenza), while the animals exposed to both clearly lost more weight (Figure 2B). Post hoc analysis using a one-way ANOVA showed that mice exposed to both HDM and influenza lost significantly more weight than saline-exposed animals (*p* < 0.02), suggesting more severe disease, while mice exposed to either HDM or influenza alone did not. In contrast to the clear negative effect on weight of animals exposed to both HDM and influenza, co-exposure did not lead to more infectious influenza viral particles (Figure 2C) or viral RNA (Figure 2D) being present compared to animals exposed to influenza only. Even though the co-exposure clearly resulted in more severe disease, this did not seem to be the result of more virus being present three days after infection.

### 2.2. Exposure to HDM and Influenza Results in Higher Serum Levels of HDM-Specific IgE and IgG1 than Either Exposure Alone

**Figure 3:**
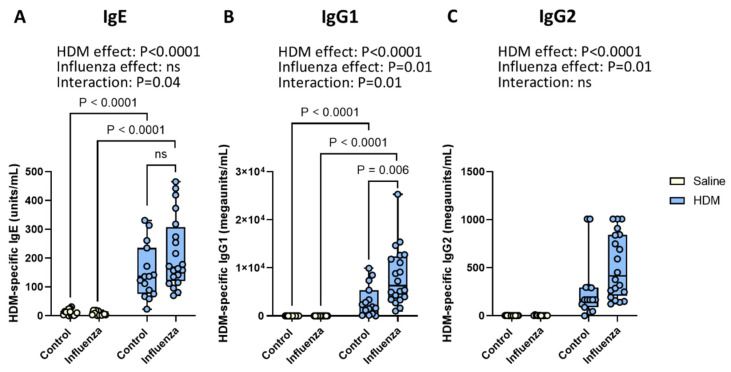
**Effects of HDM and influenza infection on HDM-specific immunoglobulin levels in serum.** Serum concentrations of HDM-specific IgE (**A**), IgG1 (**B**), and IgG2 (**C**). Data are presented as medians, with range from minimum to maximum. Groups were compared using a two-way ANOVA. When the effects of HDM and influenza virus interacted significantly, post hoc comparisons were performed using a one-way ANOVA. *p* < 0.05 was considered significant; ns, not significant.

Serum immunoglobulins, especially allergen-specific IgE, IgG1, and IgG2, are important effectors of allergic inflammation [26]. As expected, HDM exposure resulted in higher levels of HDM-specific IgE (Figure 3A), IgG1 (Figure 3B), and IgG2 (Figure 3C) as compared to non-exposed mice. Influenza infection on its own did not change HDM-specific immunoglobulins, but the two exposures did interact. The two-way ANOVA analysis indicated significant interactions between the effects of HDM and influenza infection for HDM-specific IgE and IgG1. Therefore, individual groups were compared in a post hoc analysis using a one-way ANOVA to explain this interaction. Post hoc analysis indicated that animals exposed to both HDM and influenza had significantly higher IgG1 serum levels than mice subjected to HDM alone, while IgE showed a similar but not significant pattern.

### 2.3. Exposure to HDM and Influenza Results in Fewer Neutrophils and More Alveolar Macrophages than Expected

**Figure 4:**
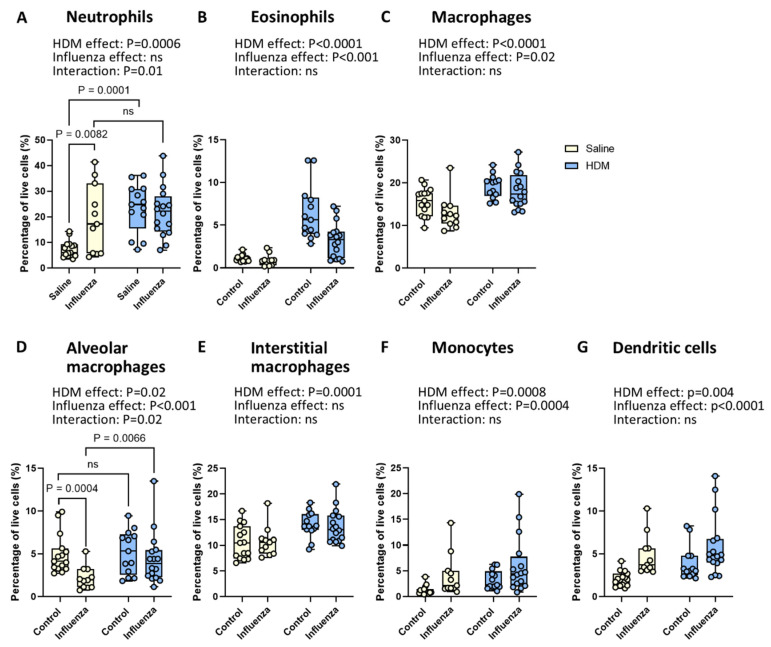
**Effects of HDM and influenza infection on myeloid cells.** Percentage of neutrophils (**A**), eosinophils (**B**), total macrophages (**C**), alveolar macrophages (**D**), interstitial macrophages (**E**), monocytes (**F**), and dendritic cells (**G**) in the lung. Data are presented as medians, with range from minimum to maximum. Groups were compared using a two-way ANOVA. When the effects of HDM and influenza virus interacted significantly, post hoc comparisons were performed using a one-way ANOVA. *p* < 0.05 was considered significant; ns, not significant.

To investigate the impact of HDM, influenza virus, and their interaction on lung inflammation in mice, we investigated the effect of HDM and influenza virus and their combination on the infiltration of myeloid cell types, i.e., neutrophils, eosinophils, total, alveolar, and interstitial macrophages, monocytes, and dendritic cells in lung tissue. HDM exposure resulted in significantly higher percentages of all myeloid cells investigated (Figure 4A–G) compared to non-exposed animals. Influenza infection, on the other hand, had more mixed effects. While more neutrophils, monocytes, and dendritic cells infiltrated lung tissue after influenza infection (Figure 4A,F,G), fewer eosinophils (Figure 4B) and macrophages (Figure 4C) were found compared to non-infected animals. The lower presence of total macrophages was driven by a specific loss of alveolar macrophage and not of interstitial macrophages (Figure 4D,E).

Interestingly, when HDM-exposed mice were also infected with the influenza virus, the effect of HDM exposure and influenza infection showed clear interactions. To interpret this interaction, we performed post hoc comparisons on the individual groups with a one-way ANOVA. For neutrophils, both HDM exposure and influenza virus infection alone resulted in significantly more neutrophils compared to control animals, while their combination did not lead to a higher percentage of neutrophils compared to either treatment alone (Figure 4A). For alveolar macrophages, influenza infection alone led to a lower percentage of alveolar macrophages compared to non-infected mice (*p* < 0.001), while exposure to HDM alone resulted in slightly more alveolar macrophages compared to saline exposure (*p* = 0.02, Figure 4D). The combination of the two exposures then resulted in significantly more alveolar macrophages compared to influenza infection alone (*p* = 0.0066). No differences in absolute numbers of myeloid cells per gram of lung tissue were found for any of the subsets (Figure S3A–G).

### 2.4. HDM Exposure Results in More, Whereas Influenza Infection Results in Fewer CD206+ Macrophages

**Figure 5:**
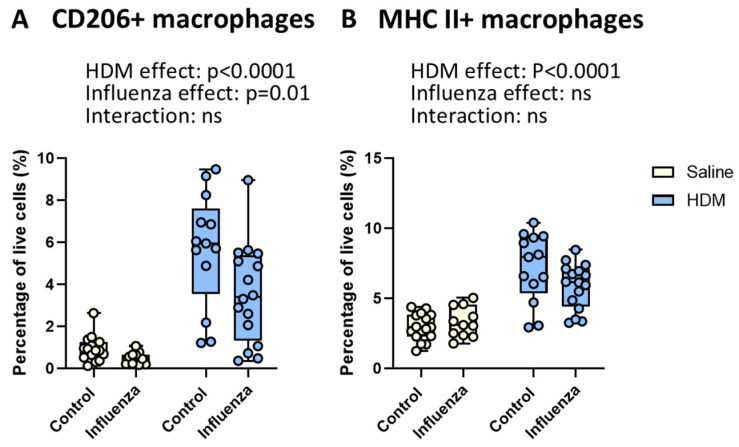
**Effects of HDM and influenza infection on macrophage polarization in lung tissue.** Percentages of CD206+ macrophages (**A**) and MHC II+ macrophages (**B**) of live cells. Data are presented as medians, with range from minimum to maximum. Groups were compared using a two-way ANOVA. *p* < 0.05 was considered significant; ns, not significant.

Our previous studies have demonstrated that the type of allergic airway inflammation induced by HDM can be influenced by macrophage polarization types [27]. Therefore, we also investigated how polarization of macrophages was affected by both HDM and influenza. Our data show that HDM exposure resulted in higher percentages of alternatively activated CD206+ macrophages (Figure 5A) and classically activated MHC II+ macrophages (Figure 5B) in lung tissue, as shown before [10,27]. Conversely, influenza infection led to a reduction in the percentage of CD206+ macrophages, while MHC II+ macrophages were not affected compared to non-infected mice. Furthermore, the effects of HDM exposure and influenza infection did not interact significantly.

### 2.5. Exposure to HDM Plus Influenza Results in Fewer Pulmonary Th1 and Regulatory T Cells and More Th2 Cells than Either Exposure Alone

**Figure 6:**
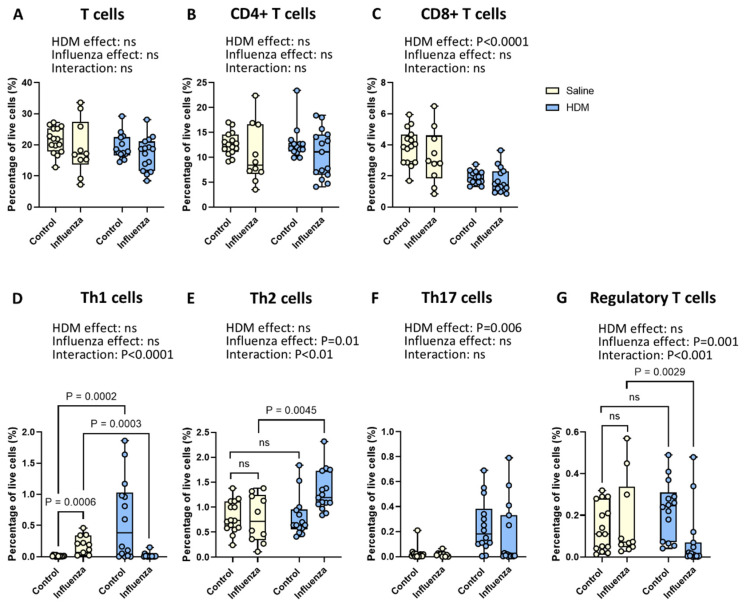
**Effects of HDM and influenza infection on lymphoid cells in the lung.** Percentage of total T cells (**A**), CD4+ T cells (**B**), CD8+ T cells (**C**), Th1 cells (**D**), Th2 cells (**E**), Th17 cells (**F**), and regulatory T cells (**G**) in mice exposed to HDM and/or influenza virus. Data are presented as medians, with range from minimum to maximum. Groups were compared using a two-way ANOVA. When the effects of HDM and influenza virus interacted significantly, post hoc comparisons were performed using a one-way ANOVA. *p* < 0.05 was considered significant; ns, not significant.

To gain further insights into the impact of HDM, influenza virus, and their interactions on lung inflammation in mice, we then examined the role of HDM and influenza virus on the infiltration of lymphoid cells in the lungs. We found no changes in the percentages of total T cells and CD4+ T helper cells for either exposure alone or their combination (Figure 6A,B), while only HDM exposure led to significantly fewer CD8+ cytotoxic T cells (Figure 6C). For the different CD4+ T cell subtypes, however, the effects of HDM exposure and influenza infection showed clear interactions for most subsets (Figure 6D–G), making it impossible to interpret the individual effects in the two-way ANOVA analysis. Therefore, individual groups were compared with a one-way ANOVA to explain this interaction. For percentages and absolute numbers of Th1 cells, both HDM exposure as well as influenza infection resulted in significantly more Th1 cells compared to control animals, while their combined use resulted in significantly lower numbers of Th1 cells compared to influenza infection alone (Figure 6D and Figure S4D). In contrast, Th2 cells were not affected by either HDM or influenza alone but were significantly higher when infection was combined with prior HDM exposure compared to infection alone (Figure 6E). With respect to Th17 cells, only HDM exposure resulted in significantly more Th17 cells in lung tissue compared to control animals (Figure 6F). For regulatory T cells, the opposite was found; only the combined exposure led to a lower percentage and absolute numbers of regulatory T cells compared to influenza alone (Figure 6G and Figure S4G). No other differences in the absolute numbers of T cell subsets were found (Figure S4A–G).

### 2.6. Th1 and Regulatory T Cell-Related Gene Expression Was Altered by the Interacting Effects of HDM and Influenza Virus Infection

**Figure 7:**
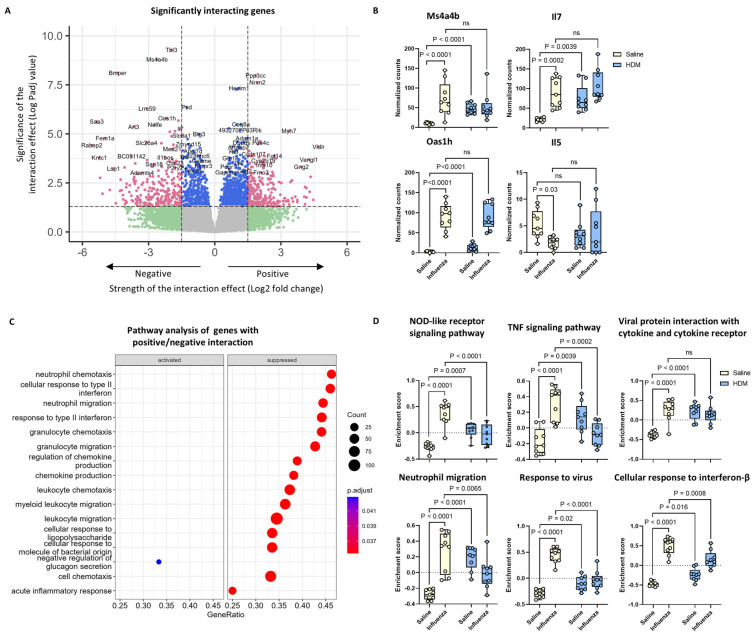
Bulk RNA sequencing analysis of lung tissue to assess the interaction between the effects of HDM and influenza virus. Volcano plot of differentially expressed genes with a positive/negative interaction between the effects of HDM and influenza exposure (*p*adj value cut off = 0.05, log2fold change cut off = ±1.5), pink dots: *p*adj < 0.05, log2fold change > |1.5|, blue dots: *p*adj < 0.05, log2fold change < |1.5|, green dots: *p*adj > 0.05, log2fold change > |1.5|, grey dots: *p*adj > 0.05, log2fold change < |1.5| (**A**), normalized counts of genes from gene set enrichment analysis (GSEA) (**B**), pathway analysis of genes with a positive/negative interaction between the effects of HDM and influenza exposure (**C**), enrichment scores of signaling pathways from gene set variation analysis (GSVA) (**D**).

To further investigate the interactions of HDM exposure and influenza virus infection, we performed bulk RNA sequence analysis on the lung tissue of mice treated with HDM, influenza, and/or their combination. In our statistical analysis, we specifically focused on the interaction effect to determine which genes and pathways could explain the different responses to influenza infection that we found in mice with established HDM-induced inflammation compared to influenza-infected mice exposed to saline. PCA analysis showed that the data from HDM-, influenza-, and co-exposed mice clustered differently from those of the non-exposed, non-infected control mice, indicating altered transcriptional characteristics from the exposures compared to control mice (Figure S6A). Both HDM exposure and influenza infection on their own resulted in many genes being differentially up- or down-regulated (Figure S5A,B). The main pathways involved in HDM exposure were ‘cell–matrix adhesion’, ‘cell junction assembly’, and ‘cell–cell junction organization’, while influenza infection resulted in ‘defense response to virus’, ‘cellular response to type II interferon’, and ‘response to interferon beta’ (Figure S5C,D). Our main interest, however, was genes specifically differentially regulated in mice exposed to both HDM and influenza compared to either exposure alone. Exposure to both HDM and influenza resulted in 133 positively and 129 negatively interacting genes (*p*adj value < 0.05 and log2fold change in expression ≥ ±1.5, Figure 7A). The top genes with a negative interaction (meaning being less up-regulated than expected in mice exposed to HDM and influenza compared to either exposure alone) included genes involved in Th1 development, such as *Ms4a4b*, *Oas1h*, and *Il7* (Figure 7B), and many others (Figure S6B). In contrast, genes related to Th2 differentiation, such as *Il4*, *Il5*, and *Irf4*, had a positive interaction (Figure 7B and Figure S6B), meaning they were expressed at higher levels than expected after HDM–influenza co-exposure compared to either exposure alone.

To obtain a better insight into the pathways regulating the genes with a negative/positive interaction, we performed a pathway analysis using gene ontology pathways. This analysis indicated that the pathways related to chemokine production and immune cell chemotaxis and migration were suppressed in the lung tissue of mice exposed to both HDM and influenza compared to either exposure alone (Figure 7C). Only one pathway appeared to be activated within the genes with significant interactions (“negative regulation of glucagon secretion”, Figure 7C). Since interaction effects are difficult to interpret, we subsequently conducted a gene set variation analysis (GSVA) for several pathways that were significantly suppressed for the interaction genes to visualize patterns (Figure 7D). This clearly visualized that several pathways related to viral infection (i.e., ‘NOD-like receptor signaling pathway’, ‘TNF signaling pathway’, ‘viral protein interaction with cytokine and cytokine receptor’, ‘neutrophil migration’, ‘response to virus’, and ‘cellular response to interferon-β’) were not induced as much in the lung tissue of mice receiving both exposures compared to mice infected with influenza alone (Figure 7D).

## 3. Discussion

In this study, we aimed to characterize response pathways to influenza virus infection in mice with pre-existing allergic inflammation to understand why asthmatics are at risk for exacerbation of disease when infected with the influenza virus. Our approach integrated data from both flow cytometry and RNA-sequencing analyses using an interaction analysis to associate cellular and transcriptomic responses [28]. By identifying different proportions of immune cells, including lymphocytes and myeloid cells, we showed that pre-existing allergic inflammation induced by HDM exposure changed the infiltration of some immune cells upon influenza virus infection. Specifically, this response was characterized by insufficient recruitment of neutrophils, fewer Th1 cells, and fewer regulatory T cells alongside more Th2 cells in mice exposed to both HDM and the influenza virus compared to mice only exposed to influenza. These changes were associated with less upregulation of genes in pathways crucial to neutrophil migration and Th1 cell development and differentiation, as well as genes in pathways associated with antiviral responses. Together, this suggests that both innate as well as adaptive immune responses in mice with pre-existing allergic inflammation and co-infected with the influenza virus are dysregulated, possibly explaining why these mice develop more severe disease after infection.

Our data show that HDM and influenza exposure together clearly contribute to more severe disease observed in our mice. This was characterized by a significant drop in body weight, which has been shown by others as well [21], an insufficient recruitment of neutrophils, and fewer Th1 cells and regulatory T cells. Neutrophils are essential for early responses to infections, contributing to pathogen clearance through phagocytosis, NETosis, and the release of antimicrobial factors [29,30]. The relatively lower presence of neutrophils may therefore indicate impaired migration and early defense mechanisms or more loss of neutrophils due to NETosis. The fact we did not find more virus particles present may suggest the latter: a more vigorous response of neutrophils with a subsequent loss of these cells.

Interestingly, adaptive responses were also affected by the combined exposure to HDM and influenza. The time point of three days after infection was too early for influenza-specific adaptive responses, but the T cells infiltrating in response to HDM were clearly affected by a subsequent infection with influenza. The observed loss in Th1 cells, important producers of interferon gamma (IFN-γ), which also plays a significant role in antiviral responses [16], may have had negative consequences for early antiviral defenses, compromising the host’s ability to control viral infection. The insufficient upregulation of antiviral-related gene pathways in mice exposed to both HDM and influenza substantiates this finding. Additionally, the increase in Th2 cells suggests a skewed immune response towards allergy-related pathways over antiviral defenses, which may be further enhanced by the lower presence of regulatory T cells failing to regulate Th2 inflammation. This shift away from Th1 infiltration towards Th2 infiltration suggests exacerbation of HDM-induced Th2 inflammation, leading to more severe disease. Our findings correlate with observations in asthma patients, who often exhibit an exaggerated Th2 response and are prone to severe exacerbations upon respiratory viral infections. Asthmatics typically have underlying airway inflammation characterized by eosinophilia and increased Th2 cytokine levels, paralleling the immune milieu seen in our HDM-exposed mice [31,32]. The impaired antiviral response and altered immune cell dynamics in these patients likely contribute to the higher morbidity and prolonged recovery times observed during influenza outbreaks [33,34,35,36,37,38].

HDM is among the most prevalent indoor environmental allergens and is known to trigger robust type 2 inflammatory responses [39]. In our study, HDM exposure alone led to allergic inflammation in mice, characterized by elevated levels of HDM-specific immunoglobulins, more Th1 cells and Th17 cells combined with infiltration of both eosinophils and neutrophils, and the presence of more CD206+ and MHCII+ macrophages compared to control mice. Surprisingly, we could not detect a higher infiltration of Th2 cells. This may be because we detected these as CD4+CD25+ cells negative for all other subset-specific markers, resulting in a less sensitive detection method. However, our method was sensitive enough to pick up the higher infiltration of Th2 cells in mice exposed to both HDM and influenza, indicating a worsening of Th2 inflammation in these mice. Additionally, HDM exposure led to a significant reduction in CD8+ T cells in lung tissue compared to saline-exposed controls. The role of CD8+ T cells in asthma is unclear; both deleterious and beneficial effects have been described [40], including suppression of IgE production. The loss of CD8+ T cells after HDM exposure may therefore have contributed to the high levels of HDM-specific IgE we found.

Influenza disease in humans is usually caused by the influenza A or B viruses [41,42]. In our mice exposed to the H3N2 influenza A virus, infection resulted in a robust Th1-driven infiltration with infiltration of neutrophils, monocytes, dendritic cells, and fewer CD206+ macrophages, as described before [22,43]. Interestingly, however, both eosinophils and alveolar macrophages were negatively affected by influenza infection, with fewer of them being present in the lung tissue of infected animals compared to controls. The depletion of alveolar macrophages after influenza infection has been described by others before [44,45], although why they disappear remains unclear. Potential reasons could include virus-induced cell death or killing by natural killer cells or CD8+ T cells. Eosinophils were shown to be an additional source of host defense against the influenza virus [46,47,48]. The group of Lutter previously showed that eosinophils are capable of rapid capture and inactivation of viruses by becoming activated and thereby contributing to antiviral responses. The lower numbers of eosinophils we found after influenza infection may indicate loss of eosinophils due to the activation and subsequent apoptosis and clearance of these cells. Alternatively, Emali et al. reported that eosinophils can migrate to the draining lymph nodes in response to influenza virus infection and play a role in presenting antigens from influenza in major histocompatibility complex class I, which is crucial for activating CD8+ T cells [47]. Therefore, the loss of eosinophils after influenza infection could also be the result of them moving to lung-draining lymph nodes to interact with CD8+ T cells.

Our study has provided valuable insights into how innate and adaptive immune responses to influenza differ on a background of asthma. Yet we must acknowledge certain limitations that may impact the interpretation and generalizability of our findings. One notable limitation is related to the fact that we only used female animals in our study. Preclinical animal studies and retrospective human studies suggest that adult females have worse outcomes from influenza than males [49,50,51,52,53,54,55,56,57,58,59]. We therefore deliberately chose the most affected sex to minimize the variance and therefore reduce the number of animals used in our study. Additionally, our flow cytometry measurements did not include B lymphocytes, which are crucial for generating allergen-specific immunoglobulins [60]. Given the increased levels of HDM-specific immunoglobulins in animals exposed to both HDM and influenza, exploring this interaction could provide deeper insights. Furthermore, including more time points after influenza virus infection could provide a more comprehensive understanding of the kinetics of antiviral responses. Lastly, while our transcriptomic and cytometric analyses identified associations, they did not establish causality, highlighting an area for further detailed studies to potentially uncover new therapeutic targets for asthma exacerbations.

Despite these limitations, the strengths of our study outweighed these limitations. We integrated findings from three independent animal experiments, significantly enhancing the reliability of our results. The large sample size added robustness to our statistical analyses, for which we employed an interaction model. This was carried out to precisely delineate the effects resulting from the combination of HDM and influenza exposure, distinguishing mere additive effects from genuine interactions. Additionally, this study is one of the few that combines detailed transcriptomic data with comprehensive immunophenotyping to explore the dynamics of respiratory virus infections in the context of allergic inflammation. Our approach provides a valuable framework for future studies aimed at unraveling complex immune interactions in disease states.

## 4. Materials and Methods

### 4.1. Animals

Female BALB/c mice were purchased from Envigo (Horst, The Netherlands). All experiments were performed using 9–11-week-old mice, which were housed in groups of 4–5 mice per cage. The mice were kept in temperature-controlled rooms maintained on a 12-h light/dark cycle and provided with ad libitum access to food, water, and cage enrichment. This study was conducted after being reviewed and approved by the Dutch National Animal Care and Use Committee in accordance with strict governmental and international guidelines for animal experimentation, under license number 105002016777.

### 4.2. Experimental Design

The data presented in this manuscript are the result of 3 independent experiments, each with 3–6 mice per experimental group. Mice were anesthetized with isoflurane and intranasally exposed to either saline or 62.5 µg of whole-culture house dust mite (HDM) extract (*Dermatophagoides pteronyssinus*, 15G10, Citeq, Groningen, The Netherlands, containing an endotoxin level of 1.65 × 10^7^ EU/gram) in saline. The animals were exposed to saline or HDM three times per week for 24 days and a single, nasal administration of 20 units of 50% tissue culture infectious dose of H3N2 influenza A virus Hong Kong (HK) X31 or saline on day 21 and were separated into four groups: saline/saline, HDM/saline, saline/influenza, and HDM/influenza, with 11–16 mice in each group when combining the three independent experiments. HK X31 virus stocks were obtained from Madin–Darby canine kidney cells infected with HK X31 virus, following the method described by Bodewes et al. [61]. On day 24, which was one day after the final HDM exposure and three days after the X31 challenge, mice were sacrificed by cardiac exsanguination.

### 4.3. Flow Cytometry

The right lung was collected for flow cytometry analysis. Preparation of single-cell suspensions from lung tissue and subsequent flow cytometry analysis followed the protocol previously described by us [62]. Two panels of antibodies were used to measure the frequencies of both lymphocytes and myeloid cells. The list of antibodies used is provided in Table S1. The gating strategy employed for the analysis of different cell types can be found in the Supplementary Materials (Figures S1 and S2). The analyzed cell types and their characteristics are summarized in Table S2.

### 4.4. Immunoglobulins Measurement

Blood was collected to measure immunoglobulin levels. HDM-specific immunoglobulin G1 (IgG1), immunoglobulin G1 (IgG2), and IgE levels in plasma were measured following the protocol provided by Citeq (Groningen, The Netherlands). Purified anti-mouse IgE-, IgG1-, and IgG2-capturing antibodies (Citeq, Groningen, The Netherlands) were incubated overnight in high-binding 96-well plates. The plates were washed with washing buffer (PBS, 0.05% *v*/*v* Tween 20) and blocked with blocking buffer (PBS, containing 1% *w*/*v* BSA) for 1 h. After washing, 100 μL of (diluted) serum was added and incubated at room temperature for 2.5 h. For IgE, samples were diluted 10–150 times; for IgG1, samples were diluted 100–30,000 times; and for IgG2a, samples were diluted 100–20,000 times depending on the sample type. After washing the plate, biotinylated HDM extract was added and incubated for 1 h. The plate was washed again and incubated with streptavidin-peroxidase for 30 min, followed by another washing step. Finally, 100 μL of substrate solution, containing 1:1 mixture of color reagent A (H_2_O_2_) and color reagent B (tetramethylbenzidine, all from R&D Systems, catalog #DY999, Minneapolis, MN, USA) was added to the plates until the positive control stained clearly, after which color development was stopped with 2N H_2_SO_4_. Absorbance was determined at 450 nm with 650 nm as a reference filter using a Synergy H1 plate reader (BioTek, Winooski, VT, USA).

### 4.5. Bulk RNA Sequencing Analysis

Total RNA was extracted from lung tissue using a Maxwell^®^ 16 LEV simplyRNA Tissue Kit (Promega, catalog #AS1280, Leiden, The Netherlands) following the manufacturer’s protocol. GenomeScan (Leiden, The Netherlands) assessed the concentration and quality of isolated RNA and used samples with enough RNA for library preparation and subsequent bulk RNA sequencing analysis. To assess the overall effect of experimental covariates and batch effects in the RNA sequencing (RNA-seq) results, principal component analysis (PCA) was performed using R package DESeq2 (version 1.42.1) in R studio (version 2023.6.0.421) [63,64]. Differential gene expression analysis was conducted, also using DESeq2. Gene set enrichment analysis (GSEA) was performed using R packages cluster profiler (version 4.10.1) to identify enriched biological pathways or functions associated with differentially expressed genes [65]. Gene set variation analysis (GSVA) was performed using R packages GSVA (version 1.50.5) to detect specific enriched pathways analysis in a sample-wise manner [66]. The raw counts of the RNA sequencing dataset can be found in Supplementary File S2. The RNA-seq dataset has also been uploaded to Gene Expression Omnibus (GEO); the exact reference number is pending.

### 4.6. Lung Virus Titration

Virus titration was performed on Madin–Darby canine kidney cells seeded in 96-well plates, as previously described [67]. The collected lung tissue was homogenized in 1 mL EpiSerf medium (twofold dilutions) and centrifuged at 1200 rpm for 10 min to collect the supernatant. These supernatants were then added to the cells. After 2 h of incubation, the medium was replaced with medium containing 7.5 μg/mL N-tosyl-L-phenylalanine chloromethyl ketone trypsin (Sigma, St. Louis, MO, USA), and the plates were incubated for another 72 h at 37 °C and 5% CO_2_. Supernatants were transferred to a V-bottom 96-well plate, and guinea pig erythrocytes (0.3% final concentration) were added. The virus titers in the lung were determined after 2 h based on the dilutions at which hemagglutination occurred. The log10 virus titer was then calculated per gram of lung tissue. The limit of detection (LoD) was determined by calculating the log10 of the first dilution, and negative values were assigned half the value of the LoD.

### 4.7. Statistical Analysis

Data are presented as medians, with a range from minimum to maximum unless otherwise specified. The normality of the data was assessed visually using quantile–quantile plots (Q–Q plots) in GraphPad Prism (version 8.0). Parametric tests were employed for normally distributed data, while for non-normally distributed data, log-transformed values were employed to attain normality. In this study, we used an unpaired *t*-test for comparisons of two independent groups and a two-way analysis of variance (ANOVA) to identify the effects of HDM exposure, influenza infection, and their combination, as described before for a study investigating interactions between smoking and influenza [28]. A two-way ANOVA analysis assessed whether HDM exposure causes significant changes compared to saline, whether influenza infection causes significant changes compared to mock infection, and whether these two exposures have a significant interaction to establish whether the combination of the two effects was more or less than a simple summation. When the effects of HDM exposure and influenza infection interacted significantly, post hoc comparisons were performed to explain the nature of the interaction. Statistically significant differences were defined as *p* < 0.05. All statistical analyses were performed using GraphPad Prism, except for RNA sequencing data. Differential gene expression analysis for RNA sequencing data was conducted using DESeq2 using treatments (HDM, influenza) and interaction term between treatment (HDM:influenza) as predictors within the model. A generalized linear model (GLM) based on the negative binomial distribution was employed to analyze the differentially expressed genes related to the effect of HDM alone, influenza alone, and an interaction effect between HDM and influenza. To correct for multiple comparisons, a Benjamini–Hochberg correction was used to control for the false discovery rate (FDR), where the adjusted *p*-value < 0.05 (*p*adj) was considered statistically significant. Genes that showed a log2fold change ≥ ±1.5 were used for further analyses.

### 4.8. AI-Assisted Writing Process 

During the writing process of this paper, the authors used ChatGPT (version 4o) to enhance language and readability. After using this tool, the authors reviewed and edited the content as needed and take full responsibility for the final content of the publication

## 5. Conclusions

In conclusion, our study demonstrates that pre-existing allergic inflammation due to HDM exposure significantly alters both innate as well as adaptive immune responses in mice co-infected with the influenza virus. The findings reveal underdeveloped antiviral responses as early as three days post-infection, which could potentially explain the heightened vulnerability of asthmatics to severe viral diseases. This study underscores the complex interplay between allergic inflammation and viral infections, highlighting the need for targeted strategies to manage influenza risks among individuals with pre-existing respiratory conditions.

## Data Availability

RNA sequencing dataset is available on GEO (GSE293097). Other data are available upon request.

## Supplementary Materials

The following supporting information can be downloaded at: https://www.mdpi.com/article/10.3390/ijms26083483/s1.

## Abbreviations

HDM	House dust mite
HK	Hong Kong
RNA-Seq	RNA sequencing
IgE	Immunoglobulin E
IgG1	Immunoglobulin G1
IgG2	Immunoglobulin G2
Th1	T helper 1
Th2	T helper 2
Th17	T helper 17
IL-4	Interleukin 4
IL-5	Interleukin 5
IL-13	Interleukin 13
PCA	Principal component analysis
GSEA	Gene set enrichment analysis
GSVA	Gene set variation analysis
GEO	Gene Expression Omnibus
LoD	Limit of detection
Q–Q plot	Quantile–quantile plot
ANOVA	Analysis of variance
MHC II	Major histocompatibility complex class II
IFN-γ	Interferon gamma

## References

[1] Global Asthma Network The Global Asthma Report 2022. www.globalasthmareport.org.

[2] Global Initiative for Asthma. Global Strategy for Asthma Management and Prevention, 2023. Updated July 2023.

[3] Castillo J.R., Peters S.P., Busse W.W. (2017). Asthma Exacerbations: Pathogenesis, Prevention, and Treatment. J. Allergy Clin. Immunol. Pract..

[4] Papadopoulos N.G., Christodoulou I., Rohde G., Agache I., Almqvist C., Bruno A., Bonini S., Bont L., Bossios A., Bousquet J. (2011). Viruses and Bacteria in Acute Asthma Exacerbations—A GA^2^ LEN-DARE Systematic Review. Allergy.

[5] Bakakos A., Sotiropoulou Z., Vontetsianos A., Zaneli S., Papaioannou A.I., Bakakos P. (2023). Epidemiology and Immunopathogenesis of Virus-Associated Asthma Exacerbations. J. Asthma Allergy.

[6] Whetstone C.E., Ranjbar M., Omer H., Cusack R.P., Gauvreau G.M. (2022). The Role of Airway Epithelial Cell Alarmins in Asthma. Cells.

[7] Wallrapp A., Riesenfeld S.J., Burkett P.R., Kuchroo V.K. (2018). Type 2 Innate Lymphoid Cells in the Induction and Resolution of Tissue Inflammation. Immunol. Rev..

[8] Harker J.A., Lloyd C.M. (2023). T Helper 2 Cells in Asthma. J. Exp. Med..

[9] Draijer C., Robbe P., Boorsma C., Hylkema M., Melgert B. (2013). Characterization of Macrophage Phenotypes in Three Murine Models of House Dust Mite-Induced Asthma. Mediat. Inflamm..

[10] Robbe P., Draijer C., Borg T., Luinge M., Timens W., Wouters I., Melgert B., Hylkema M. (2015). Distinct Macrophage Phenotypes in Allergic and Nonallergic Lung Inflammation. Am. J. Physiol. Lung Cell. Mol. Physiol..

[11] Ray A., Kolls J.K. (2017). Neutrophilic Inflammation in Asthma and Association with Disease Severity. Trends Immunol..

[12] Thomas R., Qiao S., Yang X. (2023). Th17/Treg Imbalance: Implications in Lung Inflammatory Diseases. Int. J. Mol. Sci..

[13] Gambadauro A., Galletta F., Li Pomi A., Manti S., Piedimonte G. (2024). Immune Response to Respiratory Viral Infections. Int. J. Mol. Sci..

[14] Lukhele S., Boukhaled G.M., Brooks D.G. (2019). Type I Interferon Signaling, Regulation and Gene Stimulation in Chronic Virus Infection. Semin. Immunol..

[15] Tavares L.P., Teixeira M.M., Garcia C.C. (2017). The Inflammatory Response Triggered by Influenza Virus: A Two-Edged Sword. Inflamm. Res..

[16] Draijer C., Boorsma C.E., Robbe P., Timens W., Hylkema M.N., Ten Hacken N.H., van den Berge M., Postma D.S., Melgert B.N. (2017). Human Asthma Is Characterized by More IRF5+ M1 and CD206+ M2 Macrophages and Less IL-10+ M2-like Macrophages Around Airways Compared with Healthy Airways. J. Allergy Clin. Immunol..

[17] Waithman J., Mintern J.D. (2012). Dendritic Cells and Influenza A Virus Infection. Virulence.

[18] Ravanetti L., Dijkhuis A., Sabogal Pineros Y.S., Bal S.M., Dierdorp B.S., Dekker T., Logiantara A., Adcock I.M., Rao N.L., Boon L. (2017). An Early Innate Response Underlies Severe Influenza-Induced Exacerbations of Asthma in a Novel Steroid-Insensitive and Anti-IL-5-Responsive Mouse Model. Allergy.

[19] Li B.W.S., de Bruijn M.J.W., Lukkes M., van Nimwegen M., Bergen I.M., KleinJan A., GeurtsvanKessel C.H., Andeweg A., Rimmelzwaan G.F., Hendriks R.W. (2019). T Cells and ILC2s Are Major Effector Cells in Influenza-Induced Exacerbation of Allergic Airway Inflammation in Mice. Eur. J. Immunol..

[20] Nouri H.R., Schaunaman N., Kraft M., Li L., Numata M., Chu H.W. (2023). Tollip Deficiency Exaggerates Airway Type 2 Inflammation in Mice Exposed to Allergen and Influenza A Virus: Role of the ATP/IL-33 Signaling Axis. Front. Immunol..

[21] Shahangian K., Ngan D.A., Chen H.H.R., Oh Y., Tam A., Wen J., Cheung C., Knight D.A., Dorscheid D.R., Hackett T.L. (2021). IL-4Rα Blockade Reduces Influenza-Associated Morbidity in a Murine Model of Allergic Asthma. Respir. Res..

[22] Wang H., Aloe C., McQualter J., Papanicolaou A., Vlahos R., Wilson N., Bozinovski S. (2021). G-CSFR Antagonism Reduces Mucosal Injury and Airways Fibrosis in a Virus-Dependent Model of Severe Asthma. Br. J. Pharmacol..

[23] Mori H., Parker N.S., Rodrigues D., Hulland K., Chappell D., Hincks J.S., Bright H., Evans S.M., Lamb D.J. (2013). Differences in Respiratory Syncytial Virus and Influenza Infection in a House-Dust-Mite-Induced Asthma Mouse Model: Consequences for Steroid Sensitivity. Clin. Sci..

[24] van Geffen C., Lange T., Kolahian S. (2024). Myeloid-Derived Suppressor Cells in Influenza Virus-Induced Asthma Exacerbation. Front. Immunol..

[25] Martucci C., Allen A.D., Moretto N., Bagnacani V., Fioni A., Patacchini R., Civelli M., Villetti G., Facchinetti F. (2024). CHF6297: A Novel Potent and Selective p38 MAPK Inhibitor with Robust Anti-Inflammatory Activity and Suitable for Inhaled Pulmonary Administration as Dry Powder. Front. Pharmacol..

[26] Williams J.W., Tjota M.Y., Sperling A.I. (2012). The Contribution of Allergen-Specific IgG to the Development of Th2-Mediated Airway Inflammation. J. Allergy.

[27] Draijer C., Robbe P., Boorsma C.E., Hylkema M.N., Melgert B.N. (2018). Dual Role of YM1+ M2 Macrophages in Allergic Lung Inflammation. Sci. Rep..

[28] Vlasma J.R., van der Veen T.A., de Jager M.H., Nawijn M.C., Brandsma C.A., Melgert B.N. (2024). Cigarette Smoking Prolongs Inflammation Associated with Influenza Infection and Delays Its Clearance in Mice. Am. J. Physiol. Lung Cell. Mol. Physiol..

[29] Kalil A.C., Thomas P.G. (2019). Influenza Virus-Related Critical Illness: Pathophysiology and Epidemiology. Crit. Care.

[30] Thiam H.R., Wong S.L., Wagner D.D., Waterman C.M. (2020). Cellular Mechanisms of NETosis. Annu. Rev. Cell Dev. Biol..

[31] Fahy J.V. (2015). Type 2 Inflammation in Asthma—Present in Most, Absent in Many. Nat. Rev. Immunol..

[32] Hammad H., Lambrecht B.N. (2021). The Basic Immunology of Asthma. Cell.

[33] Iikura M., Hojo M., Koketsu R., Watanabe S., Sato A., Chino H., Ro S., Masaki H., Hirashima J., Ishii S. (2015). The Importance of Bacterial and Viral Infections Associated with Adult Asthma Exacerbations in Clinical Practice. PLoS ONE.

[34] Schwarze J., Openshaw P., Jha A., Del Giacco S.R., Firinu D., Tsilochristou O., Roberts G., Selby A., Akdis C., Agache I. (2018). Influenza Burden, Prevention, and Treatment in Asthma—A Scoping Review by the EAACI Influenza in Asthma Task Force. Allergy.

[35] Bassetti M., Parisini A., Calzi A., Pallavicini F.M., Cassola G., Artioli S., Anselmo M., Pagano G., Rezza G., Viscoli C. (2011). Risk Factors for Severe Complications of the Novel Influenza A (H1N1): Analysis of Patients Hospitalized in Italy. Clin. Microbiol. Infect..

[36] Coverstone A.M., Wang L., Sumino K. (2019). Beyond Respiratory Syncytial Virus and Rhinovirus in the Pathogenesis and Exacerbation of Asthma: The Role of Metapneumovirus, Bocavirus, and Influenza Virus. Immunol. Allergy Clin. N. Am..

[37] Jain S., Kamimoto L., Bramley A.M., Schmitz A.M., Benoit S.R., Louie J., Sugerman D.E., Druckenmiller J.K., Ritger K.A., Chugh R. (2009). Hospitalized Patients with 2009 H1N1 Influenza in the United States, April–June 2009. N. Engl. J. Med..

[38] Feldman L.Y., Zhu J., To T. (2019). Estimating Age-Specific Influenza-Associated Asthma Morbidity in Ontario, Canada. Respir. Med..

[39] Jacquet A. (2013). Innate Immune Responses in House Dust Mite Allergy. ISRN Allergy.

[40] Betts R.J., Kemeny D.M. (2009). CD8+ T Cells in Asthma: Friend or Foe?. Pharmacol. Ther..

[41] Krammer F., Smith G.J.D., Fouchier R.A.M., Peiris M., Kedzierska K., Doherty P.C., Palese P., Shaw M.L., Treanor J., Webster R.G. (2018). Influenza. Nat. Rev. Dis. Primers.

[42] Centers for Disease Control and Prevention (CDC) Types of Influenza Viruses. 18 September 2024. https://www.cdc.gov/flu/about/viruses-types.html?CDC_AAref_Val=https://www.cdc.gov/flu/about/viruses/types.htm.

[43] Rodriguez-Ramirez H.G., Salinas-Carmona M.C., Barboza-Quintana O., Melo-de la Garza A., Ceceñas-Falcon L.A., Rangel-Martinez L.M., Rosas-Taraco A.G. (2014). CD206+ Cell Number Differentiates Influenza A (H1N1)pdm09 from Seasonal Influenza A Virus in Fatal Cases. Mediat. Inflamm..

[44] Ghoneim H.E., Thomas P.G., McCullers J.A. (2013). Depletion of Alveolar Macrophages During Influenza Infection Facilitates Bacterial Superinfections. J. Immunol..

[45] Duan M., Li W.C., Vlahos R., Maxwell M.J., Anderson G.P., Hibbs M.L. (2012). Distinct Macrophage Subpopulations Characterize Acute Infection and Chronic Inflammatory Lung Disease. J. Immunol..

[46] Lamichhane P.P., Samarasinghe A.E. (2019). The Role of Innate Leukocytes During Influenza Virus Infection. J. Immunol. Res..

[47] Samarasinghe A.E., Melo R.C., Duan S., LeMessurier K.S., Liedmann S., Surman S.L., Lee J.J., Hurwitz J.L., Thomas P.G., McCullers J.A. (2017). Eosinophils Promote Antiviral Immunity in Mice Infected with Influenza A Virus. J. Immunol..

[48] Sabogal Piñeros Y.S., Bal S.M., Dijkhuis A., Majoor C.J., Dierdorp B.S., Dekker T., Hoefsmit E.P., Bonta P.I., Picavet D., van der Wel N.N. (2019). Eosinophils Capture Viruses, a Capacity That Is Defective in Asthma. Allergy.

[49] Giurgea L.T., Cervantes-Medina A., Walters K.A., Scherler K., Han A., Czajkowski L.M., Baus H.A., Hunsberger S., Klein S.L., Kash J.C. (2022). Sex Differences in Influenza: The Challenge Study Experience. J. Infect. Dis..

[50] Robinson D.P., Lorenzo M.E., Jian W., Klein S.L. (2011). Elevated 17β-Estradiol Protects Females from Influenza A Virus Pathogenesis by Suppressing Inflammatory Responses. PLoS Pathog..

[51] Lorenzo M.E., Hodgson A., Robinson D.P., Kaplan J.B., Pekosz A., Klein S.L. (2011). Antibody Responses and Cross Protection Against Lethal Influenza A Viruses Differ Between the Sexes in C57BL/6 Mice. Vaccine.

[52] Vermillion M.S., Ursin R.L., Kuok D.I.T., Vom Steeg L.G., Wohlgemuth N., Hall O.J., Fink A.L., Sasse E., Nelson A., Ndeh R. (2018). Production of Amphiregulin and Recovery from Influenza Is Greater in Males Than Females. Biol. Sex Differ..

[53] Fink A.L., Engle K., Ursin R.L., Tang W.Y., Klein S.L. (2018). Biological Sex Affects Vaccine Efficacy and Protection Against Influenza in Mice. Proc. Natl. Acad. Sci. USA.

[54] Vom Steeg L.G., Dhakal S., Woldetsadik Y.A., Park H.S., Mulka K.R., Reilly E.C., Topham D.J., Klein S.L. (2020). Androgen Receptor Signaling in the Lungs Mitigates Inflammation and Improves the Outcome of Influenza in Mice. PLoS Pathog..

[55] Eshima N., Tokumaru O., Hara S., Bacal K., Korematsu S., Tabata M., Karukaya S., Yasui Y., Okabe N., Matsuishi T. (2011). Sex- and Age-Related Differences in Morbidity Rates of 2009 Pandemic Influenza A H1N1 Virus of Swine Origin in Japan. PLoS ONE.

[56] Kumar A., Zarychanski R., Pinto R., Cook D.J., Marshall J., Lacroix J., Stelfox T., Bagshaw S., Choong K., Lamontagne F. (2009). Critically Ill Patients with 2009 Influenza A(H1N1) Infection in Canada. JAMA.

[57] Wong K.C., Luscombe G.M., Hawke C. (2019). Influenza Infections in Australia 2009–2015: Is There a Combined Effect of Age and Sex on Susceptibility to Virus Subtypes?. BMC Infect. Dis..

[58] Mertz D., Lo C.K., Lytvyn L., Ortiz J.R., Loeb M., FLURISK-INVESTIGATORS (2019). Pregnancy as a Risk Factor for Severe Influenza Infection: An Individual Participant Data Meta-Analysis. BMC Infect. Dis..

[59] World Health Organization (2010). Sex, Gender and Influenza.

[60] Kliem C.V., Schaub B. (2024). The Role of Regulatory B Cells in Immune Regulation and Childhood Allergic Asthma. Mol. Cell. Pediatr..

[61] Bodewes R., Rimmelzwaan G.F., Osterhaus A.D. (2010). Animal Models for the Preclinical Evaluation of Candidate Influenza Vaccines. Expert Rev. Vaccines.

[62] Draijer C., Florez-Sampedro L., Reker-Smit C., Post E., van Dijk F., Melgert B.N. (2022). Explaining the Polarized Macrophage Pool During Murine Allergic Lung Inflammation. Front. Immunol..

[63] Love M.I., Huber W., Anders S. (2014). Moderated Estimation of Fold Change and Dispersion for RNA-Seq Data with DESeq2. Genome Biol..

[64] Zhu A., Ibrahim J.G., Love M.I. (2019). Heavy-Tailed Prior Distributions for Sequence Count Data: Removing the Noise and Preserving Large Differences. Bioinformatics.

[65] Wu T., Hu E., Xu S., Chen M., Guo P., Dai Z., Feng T., Zhou L., Tang W., Zhan L. (2021). clusterProfiler 4.0: A Universal Enrichment Tool for Interpreting Omics Data. Innovation.

[66] Hänzelmann S., Castelo R., Guinney J. (2013). GSVA: Gene Set Variation Analysis for Microarray and RNA-Seq Data. BMC Bioinform..

[67] Audouy S.A., van der Schaaf G., Hinrichs W.L., Frijlink H.W., Wilschut J., Huckriede A. (2011). Development of a Dried Influenza Whole Inactivated Virus Vaccine for Pulmonary Immunization. Vaccine.

