# Supplementary material for: Pre-Existing Allergic Inflammation Alters Both Innate and Adaptive Immune Responses in Mice Co-Infected with Influenza Virus
Source: Int J Mol Sci. 2025 Apr 8;26(8):3483. doi: 10.3390/ijms26083483 (PMC12027099; doi:10.3390/ijms26083483)
Supplement: Supplementary file 1 [file ijms-26-03483-s001.zip › ijms-3459707-supplementary.pdf]

Supplementary Materials

Pre-Existing Allergic Inflammation Alters Both Innate and Adaptive Immune Responses in Mice Co-Infected with Influenza Virus

Dan Li [1,2], T. Anienke van der Veen [1,2], Linsey E. S. de Groot [3], Marina H. de Jager [1], Andy Lan [1], Hoeke A. Baarsma [1,2], René Lutter [3], Kees van der Graaf [4], Reinoud Gosens [1,2], Martina Schmidt [1,2] and Barbro N. Melgert [1,2,*]

[1] Groningen Research Institute of Pharmacy, Department of Molecular Pharmacology, University of Groningen, 9713 AV Groningen, The Netherlands
[2] Groningen Research Institute for Asthma and COPD (GRIAC), University Medical Center Groningen, University of Groningen, 9713 AV Groningen, The Netherlands
[3] Department of Respiratory Medicine, Amsterdam UMC, University of Amsterdam, 1105 AZ Amsterdam, The Netherlands
[4] Citeq Biologics BV, 9726 GN Groningen, The Netherlands; vandergraaf@citeq.com
* Correspondence: b.n.melgert@rug.nl

Table S1. Antibodies for flow cytometry analysis

Antibodies	Dilution	Company
**Myeloid cells panel**		
Live/dead dye eFluor 506	1:2000	eBioscience, #65-0866-14
Anti-GR1-Brilliant Violet711	1:200	BD Biosciences, #563979
Anti-CD11C-Brilliant Violet785	1:200	BD Biosciences, #563735
Anti-CD68-PerCP/ Cyanine5.5	1:100	Biolegend, #137009
Anti-CD64-PE/Cyanine7	1:200	Biolegend, #139314
Anti-CD206-Alexa Fluor647	1:200	Biolegend, #141712
Anti-CD11b-PE	1:200	Biolegend, #101207
Anti-Ly6C-Alexa Fluor488	1:200	Biolegend, #128022
Anti-MHCII-APC/Cyanine7	1:100	Biolegend # 107627
Anti-CD170 (Siglec F)-Alexa Fluor700	1:200	eBioscience, #56-1702-80
**T cell panel**		
Live/dead dye eFluor 506	1:2000	eBioscience, #65-0866-14
Anti-RORγt-Alexa Fluor647	1:100	BD Biosciences, #562682
Anti-CD68-PerCP/Cyanine5.5	1:100	Biolegend, #137009
Anti-CD4-PE/Cyanine7	1:100	Biolegend, #100422
Anti-CD8-Alexa Fluor700	1:100	Biolegend, #100730
Anti-Tbet-Brilliant Violet711	1:50	Biolegend, #644819
Anti-Foxp3-FITC	1:100	eBioscience, #11-5773-82

Table S2. Cell types and their characteristics

**Cell type**	**Characteristics**
**Live cells**	Negative for Live/dead dye eFluor 506
**Myeloid cells panel**	
Eosinophils	CD68-GR1-CD11b+
Neutrophils	CD68-GR1+CD11b+
Monocytes	CD68-GR1-CD11b+CD11c-
Dendritic cells	CD68-GR1-CD11b+CD11c+
Macrophages	CD68+GR1-
Interstitial macrophages	CD68+GR1-CD11b+CD11c-
Alveolar macrophages	CD68+ GR1-CD11b-CD11c+
$MHCII^{high}$ macrophages	CD68+GR1-MHCII+
$CD206^{high}$ macrophages	CD68+GR1-CD206+
**T cell panel**	
T helper cells	CD3+CD25+CD4+CD8-
Th1 cells	CD3+CD4+CD25+Tbet+
Th2 cells	CD3+CD25+CD4+Foxp3-Rorγt-Tbet-
Th17 cells	CD3+CD25+CD4+Rorγt+
Regulatory T cells	CD3+CD25+CD4+Foxp3+
Cytotoxic T cells	CD3+CD25+CD4-CD8+

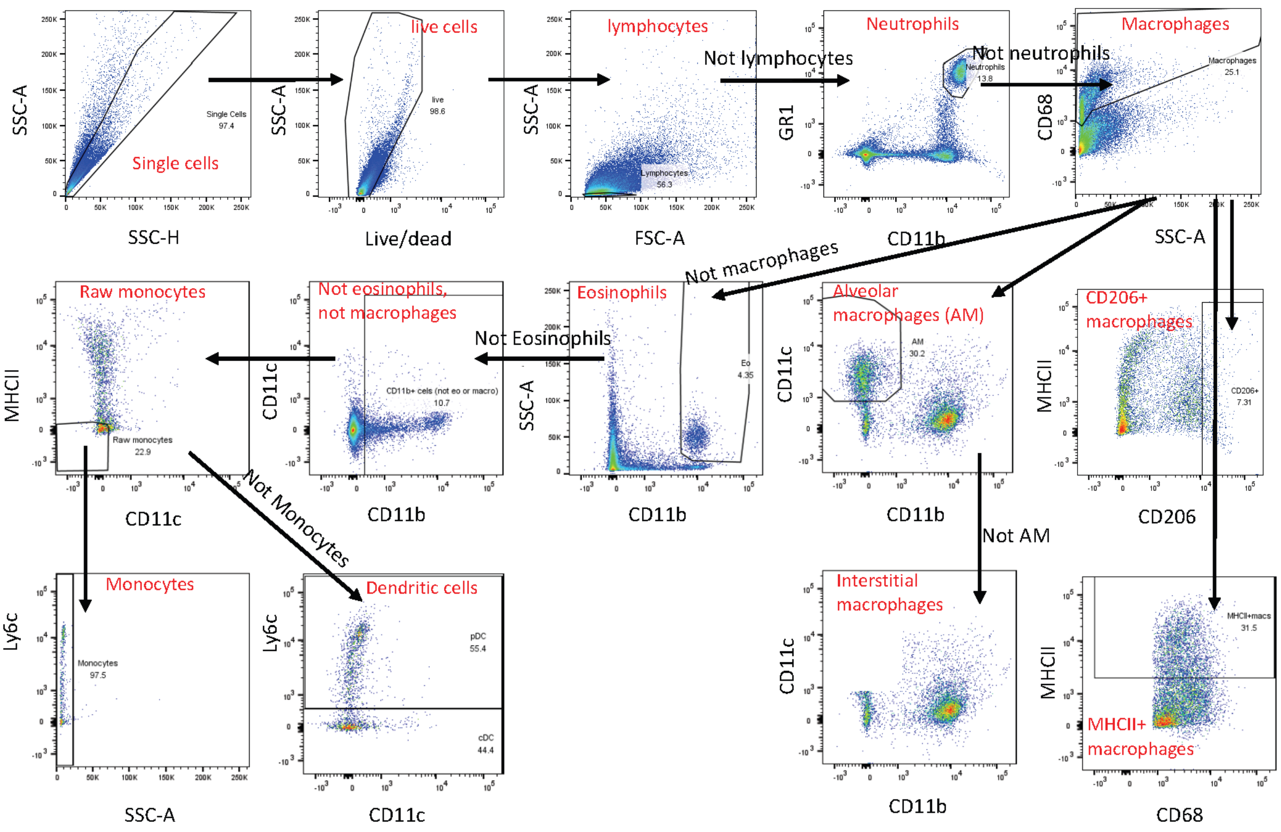

Figure S1. Gating strategy for myeloid cells.

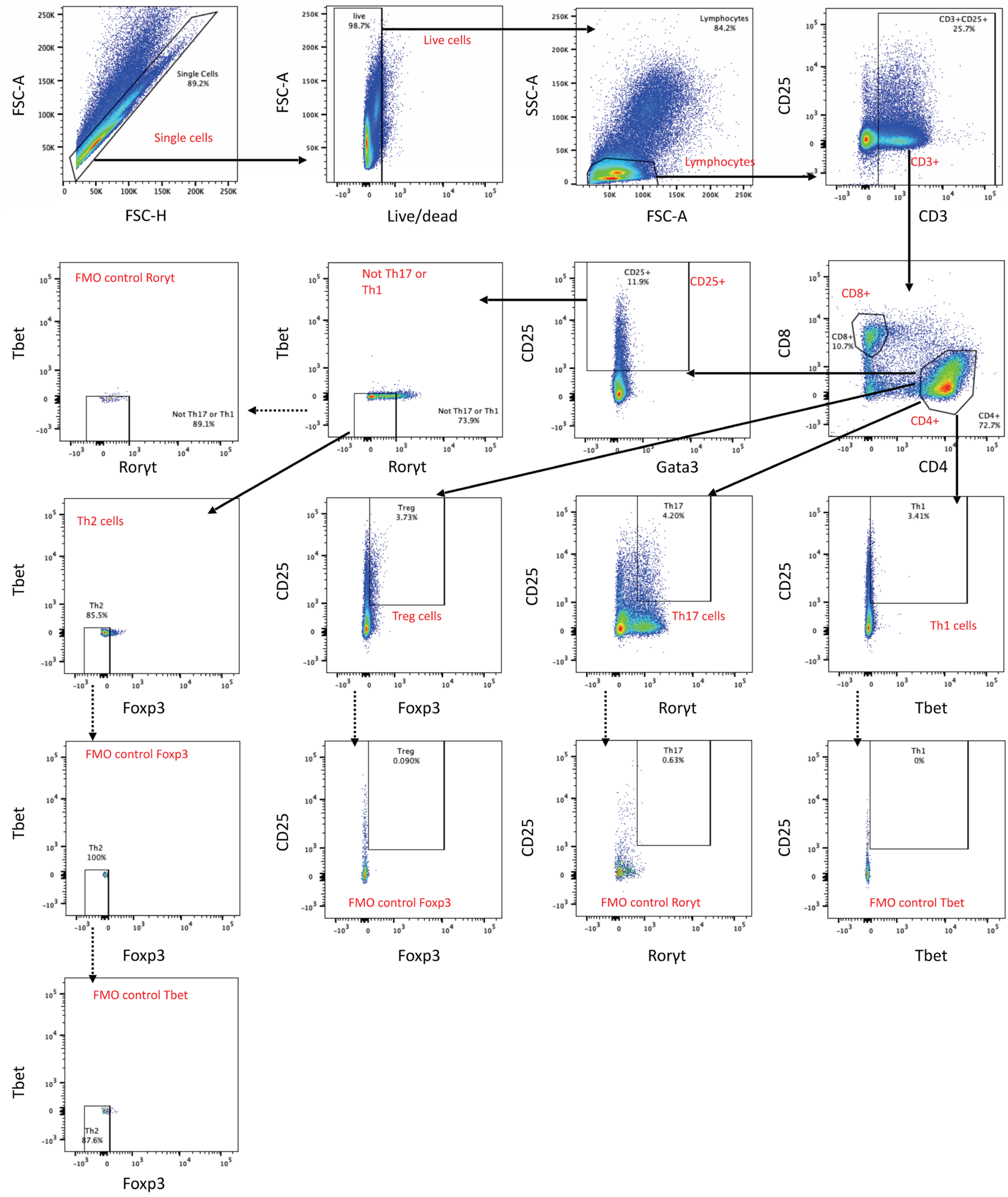

Figure S2. Gating strategy for lymphoid cells.

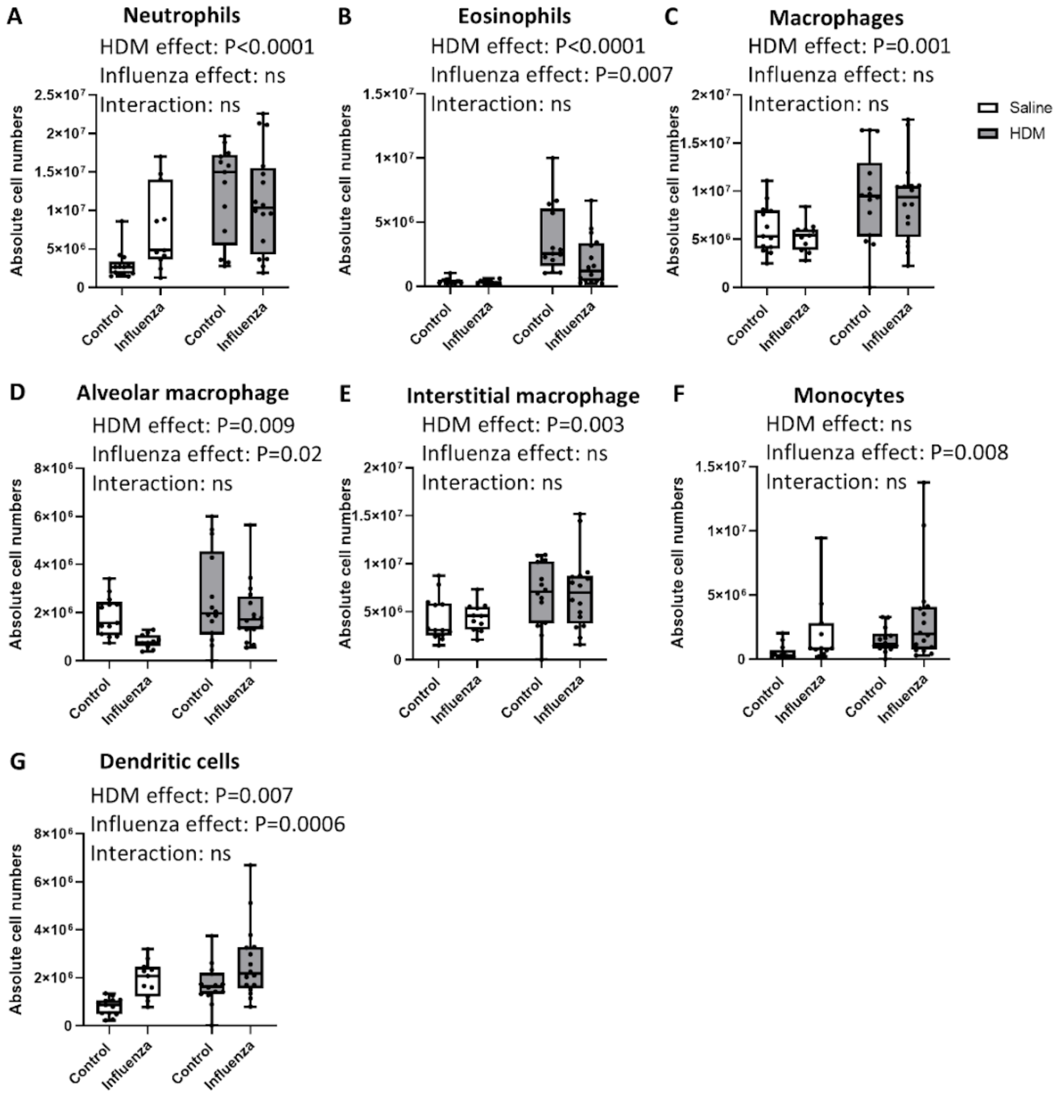

Figure S3. Absolute cell numbers of myeloid cells. Total cell numbers per gram of lung tissue of neutrophils (A), eosinophils (B), total macrophages (C), alveolar macrophages (D), interstitial macrophages (E), monocytes (F) and dendritic cells (G) in the lung. Data are presented as medians with range from minimum to maximum. Groups were compared using a two-way ANOVA.

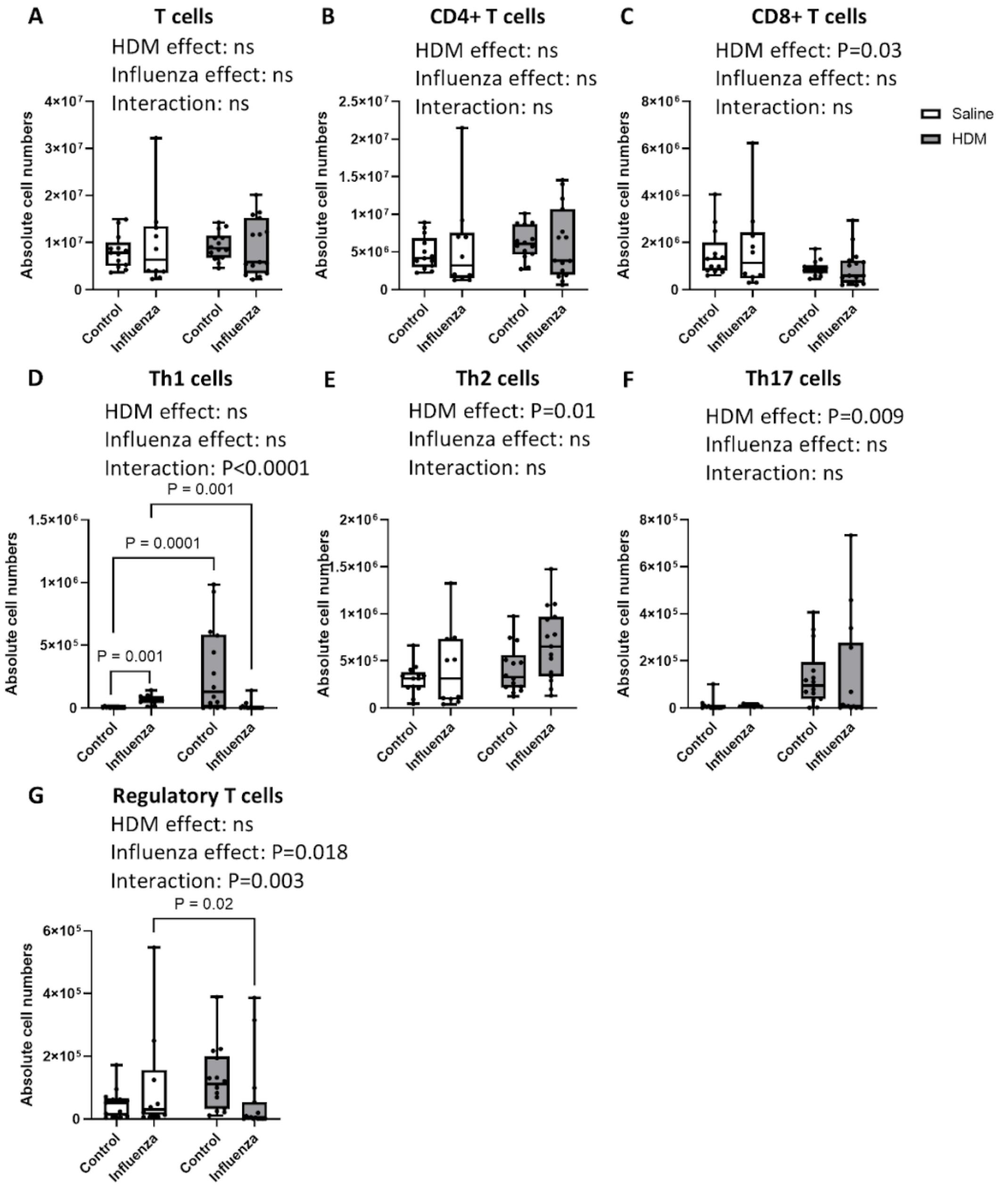

Figure S4. Absolute cell numbers of lymphoid cells. Total cell numbers per gram of lung tissue of total T cells (A), CD4+ T cells (B), CD8+ T cells (C), Th1 cells (D), Th2 cells (E), Th17 cells (F), and regulatory T cells (G) in mice exposed to HDM and/ or influenza virus. Data are presented as medians with range from minimum to maximum. Groups were compared using a two-way ANOVA. When the effects of HDM and influenza virus interacted significantly, post hoc comparisons were performed using a one-way ANOVA. $P<0.05$ was considered significant; ns, not significant.

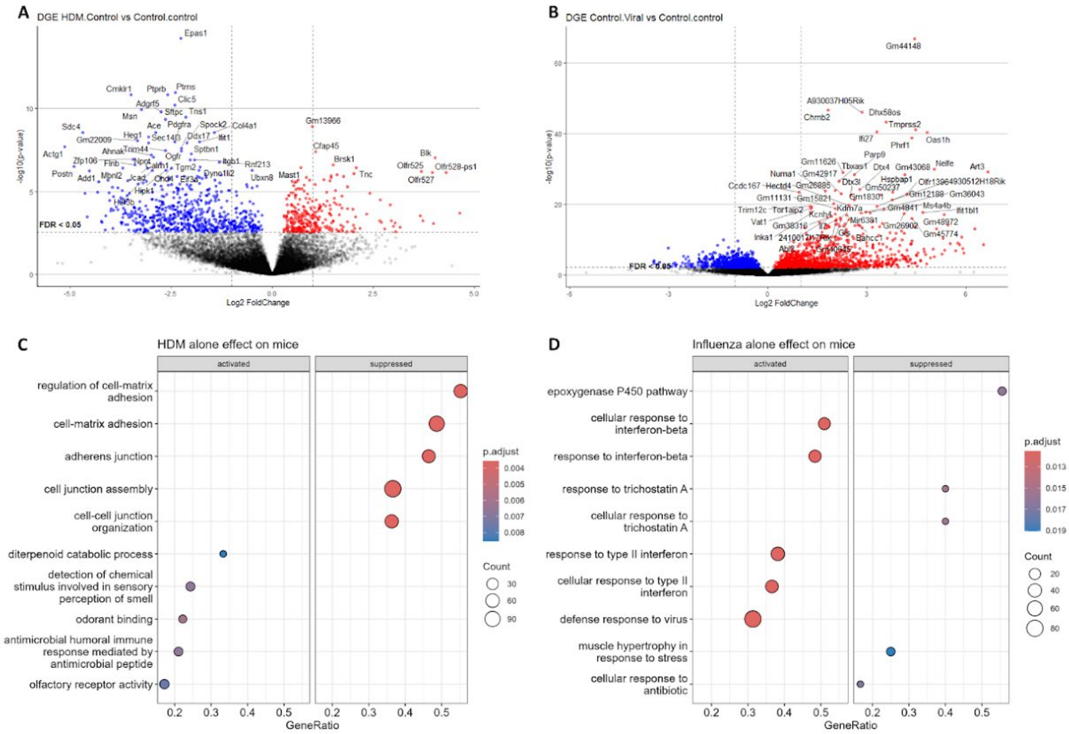

Figure S5. Bulk RNA sequence analysis of lung tissue of the effects of exposure to HDM alone or influenza virus alone. Volcano plots of the effects of HDM alone (A) or influenza virus alone (B), gene set enrichment analysis for the effects of HDM alone (C) or influenza virus alone (D) from bulk RNA sequence analysis.

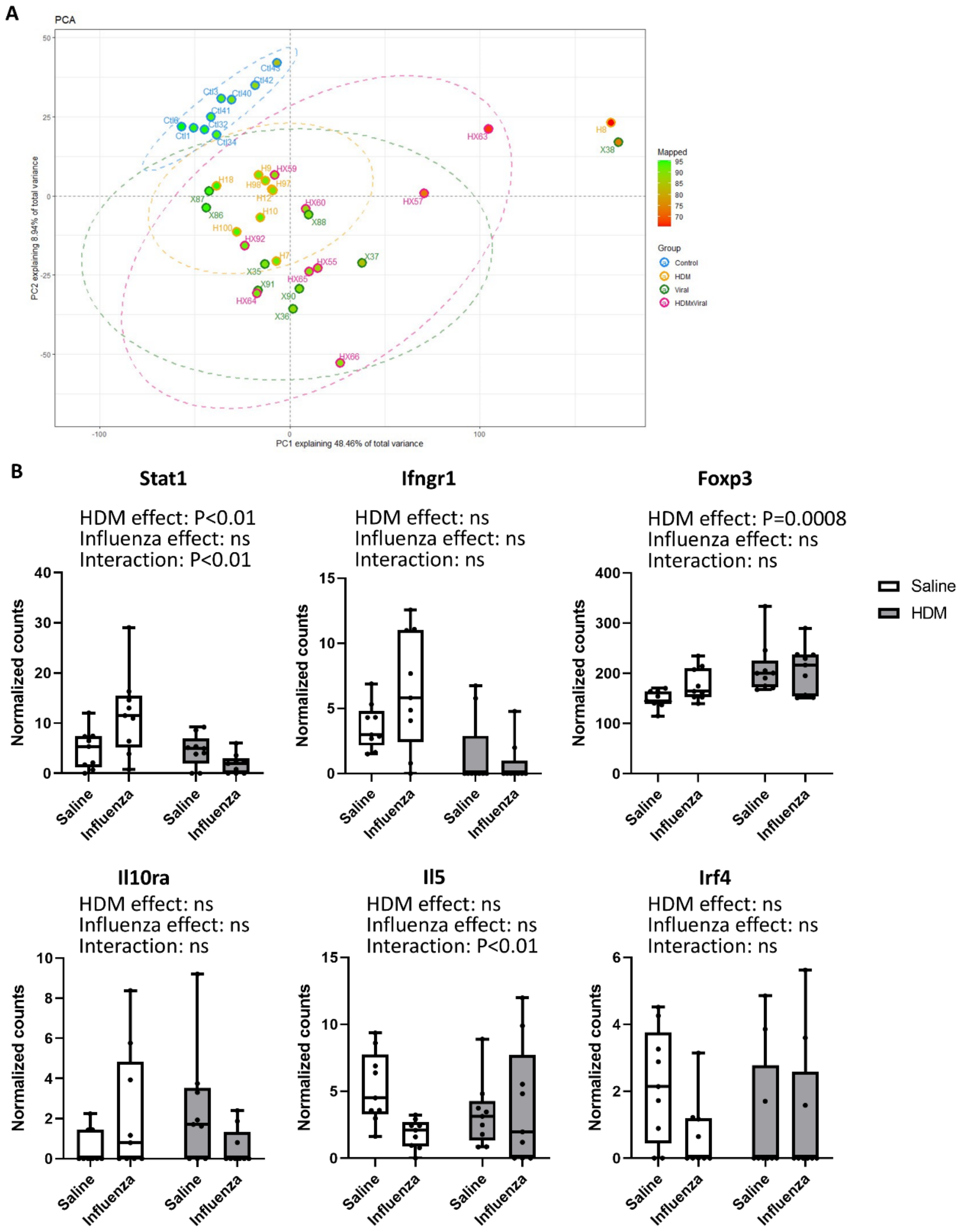

Figure S6. Bulk RNA sequence analysis of lung tissue for interactions between the effects of HDM and influenza virus. Principal component analysis (A) and plotted normalized counts of targeted genes (B).
